# Circulating Th17/Treg as a promising biomarker for patients with rheumatoid arthritis in indicating comorbidity with atherosclerotic cardiovascular disease

**DOI:** 10.1002/clc.24065

**Published:** 2023-09-04

**Authors:** Hongxuan Fan, Jianqi Zhao, Shaobin Mao, Yongle Wang, Miao Wang, Xiaosu Song, Gaizhen Liu, Caihong Wang, Xin Wang, Bin Liang

**Affiliations:** ^1^ Department of Cardiology The Second Hospital of Shanxi Medical University Taiyuan Shanxi China; ^2^ Department of Neurology The First Hospital of Shanxi Medical University Taiyuan Shanxi China; ^3^ Department of Rheumatology The Second Hospital of Shanxi Medical University Taiyuan Shanxi China

**Keywords:** biomarkers, cardiovascular, lymphocytes, rheumatoid arthritis, T cells, Th17/Treg

## Abstract

**Background:**

Immune and inflammatory responses have a pivotal role in the pathogenesis of rheumatoid arthritis (RA) and atherosclerotic cardiovascular disease (ASCVD). This study aims to explore the change of peripheral lymphocytes, especially the absolute and relative changes in peripheral T cells in RA patients with and without ASCVD.

**Hypothesis:**

The changes in the lymphocyte subsets were assessed to provide a novel insight in diagnosing and preventing ASCVD in patients with RA.

**Methods:**

A propensity score matching system (1:1) was conducted to perform a matched case–control study with 169 pairs RA‐ASCVD and RA participants. Univariate and multivariate analyses were performed to determine the association between peripheral lymphocytes and RA‐ASCVD.

**Result:**

Multivariate logistic regression analysis demonstrated that Th17 cell absolute, Th17 cell Ratio, Th17/Treg were associated with a significantly higher risk of ASCVD after model adjustment. Then we focused on Th17/Treg, multivariate logistic analyses in tri‐sectional Th17/Treg groups showed that the odds of ASCVD is gradually increasing with Th17/Treg rank's rising after model adjustment. Finally, the restricted cubic spline of Th17/Treg and odds ratio of RA‐ASCVD was conducted. Interestingly, we found a critical point of Th17/Treg (critical point = 0.2399). Th17/Treg shows a protective role in the odds of ASCVD when Th17/Treg < 0.2399. With smaller Th17/Treg, the protective efficiency is more obvious when Th17/Treg < 0.2399.

**Conclusions:**

Our study suggested that increasing absolute and percentage of Th17 cells in the peripheral blood of patients with RA was associated with the development of ASCVD. And Th17/Treg may be a promising biomarker for patients with RA in indicating comorbidity with ASCVD.

## Key Messages

Absolute and percentage of Th17 cells in peripheral blood of patients with rheumatoid arthritis‐atherosclerotic cardiovascular disease (RA‐ASCVD) is significantly higher. Elevated Th17/Treg may be a potential biomarker for RA patients in predicting comorbidity with ASCVD. The imbalance of Th17/Treg may be involved in the pathogenesis of ASCVD in rheumatoid arthritis. Downregulating Th17/Treg could be potential therapeutic targets for preventing RA patients from ASCVD.

## Graphical abstract

A propensity score matching system (1:1) was conducted to perform a matched case–control study with 169 pairs rheumatoid arthritis‐atherosclerotic cardiovascular disease (RA‐ASCVD) and RA participants. Multivariate logistic regression analysis demonstrated that Th17/Treg were associated with a significantly higher risk of ASCVD after model adjustment. Interestingly, we found a critical point of Th17/Treg (critical point = 0.2399). Th17/Treg shows a protective role in the odds of ASCVD when Th17/Treg < 0.2399.

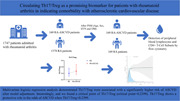

## 1 INTRODUCTION

Rheumatoid arthritis (RA) is a chronic, autoimmune, inflammatory joint disease, which can cause cartilage and bone damage, and persistent or recurrent episodes of musculoskeletal pain, swelling, and stiffness can decrease quality of life and even cause disability.^1^ Nevertheless, RA‐associated inflammatory reaction not only damages the joint and synovium, but also causes lesions on multiple tissues such as vessels, myocardium, and autonomic nerves and so on, which leads to the occurrence of multisystem diseases. Some patients with RA may present with disease manifestations in other organs. A growing number of studies show that RA patients have a higher risk of interstitial lung disease,^2^ cardiovascular disease (CVD),^3^ and even atrial fibrillation (AF),^4^ mortality than the general population. CVD resulting from chronic inflammation plays a vital role in increasing the economic burden and mortality of RA patients.^5^ Although the comorbidity of RA and CVD is widely reported, the potential relationship between RA and CVD has not been well studied.

CVD is the leading cause of death in China, accounting for 40% of deaths in the Chinese population. Internationally, China and India have the highest burdens of CVD.^6^ Our knowledge is that atherosclerosis is the culprit of CVD. Atherosclerosis contributes directly to ischemic heart disease, peripheral arterial disease and stroke due to the build‐up of plaques within blood vessels. Angina pectoris, the most common clinical syndrome of CVD, is characterized by paroxysmal chest pain or chest discomfort caused by insufficient blood supply to the coronary artery and rapid temporary ischemia and hypoxia of the myocardium.^7^ A combination of altered hemodynamics, progressive myocardial fibrosis, heart failure, and sudden cardiac death contributes to CVD morbidity and mortality. Thus, CVD is a major burden on the healthcare systems.^8^ However, the pathophysiology of atherosclerosis is complex and not fully understood. Research indicates that its occurrence and progression are related to immunity.^9^

In RA, various antibodies are produced and T helper cell components are imbalanced, leading to adverse effects on multiple body systems. And immune disorder is involved in the occurrence and development of atherosclerosis. Therefore, we speculate that in atherosclerotic cardiovascular disease (RA‐ASCVD) population, it is the disorder of the immune system in RA patients that accelerates the occurrence and development of atherosclerosis. However, the immune functions of T helper cells in the peripheral blood are unclear in the development of RA‐ASCVD. A systemic understanding of the clinical features of patients with RA‐ASCVD is urgently needed to protect susceptible RA patients. This present study was therefore designed to establish robust evidence for the association of elevated Th17 cells in peripheral blood and RA‐ASCVD.

## 2 MATERIALS AND METHODS

### 2.1 Patient information

This is a retrospective case–control study used propensity score matching (PSM) to control for confounding factors. By taking a medical history, we divided the patients admitted with RA into RA group and RA‐ASCVD group. After the grouping is determined and before receiving a new drug regimen, blood sampling will be collected after 2–24 h after the participant's admission to the hospital, and blood samples will be stored for the next analytical processing. We finally included 169 RA patients with CVD and 169 RA patients without CVD determined by PSM (1:1). All of the 338 patients required fulfillment of the RA classification standard of the American Society of Rheumatology in 1987.^10^ The inclusion criteria comprised morning stiffness, arthritis in three or more joints, hand joints, symmetrical arthritis for at least 6 weeks, rheumatoid nodules, and changes in the serum rheumatoid factor and radiographical data. CVD was a broad term that defined as s group of dysfunctions including coronary atherosclerosis heart disease, myocardial infarction, cerebral atherosclerosis, cerebrovascular infarction, and peripheral vascular disease. Patients had a clinical diagnosis of CVD according to symptoms, electrocardiographs, echocardiograms, cardiac multidetector row computed tomography, vascular ultrasound, and cranial computed tomography without regard to the presence or absence of specific criterion. The exclusion criteria comprised valvular disease and/or mechanical heart valves, AF, congenital heart disease, cardiomyopathy, active infection, malignant tumors, anemia, hematopoietic system abnormality, severe lung, liver and renal thyroid or other organ dysfunction, additional exclusion criteria included primary biliary cirrhosis, hashimoto's thyroiditis, ulcerative colitis, nephrotic syndrome and other rheumatic diseases. In addition, we traced the medication history of each participant before the blood draw through the hospital medical record system. Regular use of hormones or antirheumatic drugs or both of two drugs by our participants for the 3 months before the immune cell analysis were defined as regular medication use. All of the 338 patients from the Department of Rheumatology, the Second Affiliated Hospital of Shanxi medical University from December 2015 to October 2021. The study was approved by the ethics committee of the Second Hospital of Shanxi Medical University. ID (2021): YX No. 035.

### 2.2 Blood specimen and experimental reagents

The blood specimens we use are fasting blood taken from hospitalized patients. Typically, this blood is taken from a peripheral vein, which will then be immediately sent to the rheumatology lab. EDTA or heparin is often used as the anticoagulant. When it comes to the experimental reagents, Absolute count—TruCount™ Test tube (containing a known number of beans); FACS hemolysin solution (10×, diluted to 1× with distilled water before use); Monoclonal antibodies against CD3/CD8/CD45/CD4, CD3/CD16 + 56/CD45/CD19 were employed for the lymphocyte assay and Stimulin; Ionomycin; Golgi blocker; Phorbol ester; Fetal bovine serum; RPMI1640 medium (sigma Aldrich Corp.); Multitest CD3‐fluorescein isothiocyanate (FITC)/CD8‐PE/CD45‐PercP/CD4‐APC kits; Multitest CD3‐FITC/CD16 + 56‐PE/CD45‐PercP/CD19‐APC kits; Monoclonal antibodies to CD4‐FITC, IL‐4‐PE, IFN‐c‐APC, IL‐17‐PE, CD25‐APC, and FOXP3‐PE (Becton Dickinson and Co.) for the CD4^+^ T cell subpopulation assay. Additionally, a FACS Calibur flow cytometer supplied by BD, USA was used for the flow cytometry test.

### 2.3 Detection of peripheral blood lymphocytes by flow cytometry

In separate TruCount tubes, 50 μL of totally anticoagulated blood was combined with 20 μL of CD3/CD8/CD45/CD4 antibody and 20 μL of CD3/CD16 + 56/CD45/CD19 antibody for 15–20 min at room temperature and shielded from light for lymphocyte detection. Each tube's contents were then combined with 450 μL of XFAC hemolysin, incubated for 15 min at room temperature, shielded from light, and identified 24 h later by flow cytometry to determine the percentage and precise number of lymphocyte subsets. The surface antigen distribution was CD3^+^CD19^−^ for T lymphocytes, CD3^−^CD19^+^ for B lymphocytes, and CD4^+^ T lymphocytes and CD8^+^ T lymphocytes were CD3^+^CD4^+^ versus CD3^+^CD8^+^, respectively, and NK cells were CD3^−^CD16^+^CD56^+^. It is important to note that for the test, we extracted 15 000 cells using the MultiSET™ software (Becton Dickinson and Co.).

### 2.4 Detection of CD4^+^ T−cell subsets by flow cytometry

Briefly, anticoagulated blood was incubated under our prescribed conditions, and the cultured cells were then stained for intracellular cytokines and cell membrane surface antigens. Flow cytometry was used to detect IFN‐, IL‐4, and IL‐17 in CD4^+^ cells, and CD4^+^CD25^+^Foxp3^+^Treg cells also required to be on flow cytometry, to monitor the cellular balance of Th17/Treg.

### 2.5 Culture and detection of Th1/Th2/Th17 cells

A total of 10 μL of phorbol ester (final concentration of 30 ng/mL), 10 μL of ionomycin (final concentration of 750 ng/mL), 1 μL of BD golgistop™ (Becton Dickinson et al.) and 80 μL of anticoagulated blood were incubated in a CO_2_ atmosphere at 37°C for 5 h. After dividing the cultivated cells into tubes A and B, FITC antihuman CD4^+^ antibody was added to each tube, and the tubes were then left to incubate for 30 min at room temperature and in the dark. Next, 1 mL of freshly prepared fixation/permeabilization solution was added separately, vortexed and mixed well, and then incubated for 30 min at 4°C in the dark. At this point, interferon (IFN)‐c‐APC and interleukin (IL)‐4‐pe were then added to tube A and antihuman IL‐17‐PE was added to tube B, and both tubes were left at room temperature and shielded from light for 30 min. Following a phosphate buffered saline (PBS) wash, the contents were examined by FACSCalcium flow cytometry (Becton Dickinson et al.).

### 2.6 Culture and detection of treg cells

Briefly, anticoagulated blood (80 μL) was incubated with CD4‐FITC and CD25‐APC for 30 min at room temperature in the dark, then with 1 mL of freshly made fixative/permeabilization solution for 30 min at 4°C in the dark. Finally, antihuman forkhead box P3 (FOXP3) antibody was stained for 30 min at room temperature in the dark, washed with PBS, and flow cytometry was used to detect the results.

### 2.7 Flow cytometry assays

Using flow cytometry, cells within 24 h were identified, and Th (CD4^+^) cells were differentiated based on the forward scatter and lateral scatter values. With the use of Becton Dickinson and Co.'s cellquest™ software, we may examine the relative ratios. The ratio (%) of the total number of positive cells and CD4^+^ T cells in each subgroup was multiplied by the total number of CD4^+^ T cells/mL to determine the total number of cells in each subgroup.

### 2.8 Propensity score‐matched analysis

PSM was applied to minimize bias in the selection of patients who had RA with and without ASCVD. Propensity scores were constructed for each participant using the confounding categorical variables that influenced the incidence of ASCVD: age, sex, hypertension, and diabetes mellitus. We applied optimal PSM to generate a cohort of 169 matched pairs.

### 2.9 Statistical analysis

The normality of continuous variable distribution was evaluated by Shapiro–Wilk test. Normally distributed data are represented by the mean of standard deviation (SD). Nonnormally distributed data are represented as median with quartile range. Categorical variables are described as frequency and ratio (%). The normal distribution values of RA group with or without ASCVD were compared by unpaired Student *t* test. Comparisons Wilcoxon Test was used to analyze the difference of two independent groups of patients' variables. The nonnormal distribution values of RA group with or without ASCVD were compared by Wilcoxon rank sum test or Mann–Whitney test. First, the potential predictors of ASCVD were studied by univariate logistic regression and then multivariate analysis was carried out to determine the independent predictors and their power. The restricted cubic spline analysis was conducted to figure out the association between potential factors and odds ratio (OR) of RA‐ASCVD. All values with *p* < .05 were statistically significant.

## 3 RESULTS

### 3.1 General features of patients with RA and ASCVD

Supporting Information: Figure 1 is the flow chart of screening and enrollment, which eventually included 169 RA‐ASCVD cases and 169 RA controls. The 169 RA‐ASCVD cases were defined the study group and the 169 RA controls without ASCVD were defined the control group. There were significantly different in age, gender, hypertension, and diabetes mellitus between the two groups before PSM (*p* < .05). Then the optimal tendency matching method of PSM was utilized and the matching ratio was 1:1. Finally, 169 pairs were successfully matched. There were not significantly different in age, gender, hypertension, and diabetes mellitus after PSM (*p* > .05) Supporting Information: Table 1. Table 1 displays the characteristics of patients with RA with and without ASCVD after PSM. In the RA‐ASCVD group, there was a higher proportion of smoking, drinking alcohol history and higher weight and BMI (*p* < .05). However, there were no significant differences between the two groups in various inflammatory indexes and blood lipid values (*p* > .05). In terms of drug use in these two groups, the results showed that there was a higher proportion of nonsteroidal antiinflammatory drugs (NSAIDs) use, statin use and Beta‐blockers use in RA‐ASCVD group (*p* < .05).

**Table 1:** The demographic information and clinical data in patients with RA and RA‐ASCVD.

Variables	Total (*n* = 338)	RA group (*n* = 169)	RA‐ASCVD group (*n* = 169)	*p* Value
Age	64.3 ± 8.3	64.7 ± 8.2	63.9 ± 8.4	.38
Gender				.579
Female	202 (59.8)	104 (61.5)	98 (58)	
Male	136 (40.2)	65 (38.5)	71 (42)	
Hypertension				.743
No	156 (46.2)	80 (47.3)	76 (45)	
Yes	182 (53.8)	89 (52.7)	93 (55)	
Diabetes mellitus				1
No	256 (75.7)	128 (75.7)	128 (75.7)	
Yes	82 (24.3)	41 (24.3)	41 (24.3)	
Smoking history				.033
No	235 (69.5)	127 (75.1)	108 (63.9)	
Yes	103 (30.5)	42 (24.9)	61 (36.1)	
Drinking alcohol history				.05
No	286 (84.6)	150 (88.8)	136 (80.5)	
Yes	52 (15.4)	19 (11.2)	33 (19.5)	
Height	162.3 ± 7.7	162.7 ± 7.8	161.9 ± 7.7	.358
Weight	62.6 ± 10.4	61.4 ± 10.3	63.8 ± 10.4	.03
BMI	23.7 ± 3.5	23.2 ± 3.6	24.3 ± 3.3	.003
RF	80.0 (20.0, 320.0)	80.0 (20.0, 205.7)	82.5 (20.0, 320.0)	.405
CCP	418.8 (110.3, 996.9)	342.3 (100.8, 935.5)	443.8 (131.2, 1037.0)	.335
ESR	46.0 (24.0, 86.2)	42.0 (24.0, 83.0)	50.0 (24.0, 88.0)	.546
CRP	13.8 (4.7, 43.9)	13.0 (5.2, 38.7)	15.3 (4.2, 47.0)	.689
TG	1.1 (0.9, 1.6)	1.1 (0.8, 1.5)	1.1 (0.9, 1.6)	.171
HDL	1.2 ± 0.3	1.2 ± 0.3	1.2 ± 0.4	.923
LDL	2.4 ± 0.8	2.4 ± 0.7	2.4 ± 0.8	.759
ALT	15.9 (11.9, 23.8)	15.5 (12.3, 21.6)	16.8 (11.3, 25.0)	.838
AST	17.8 (14.2, 22.9)	17.6 (14.2, 21.5)	18.2 (14.5, 24.5)	.248
Cr	54.0 (48.0, 63.0)	54.0 (46.0, 63.0)	55.0 (49.0, 64.0)	.128
PLT	282.2 ± 107.8	292.5 ± 117.8	271.9 ± 96.1	.079
DD	539.0 (256.0, 1060.0)	571.0 (272.0, 1129.0)	526.0 (246.0, 975.0	.303
Fib	4.0 (3.2, 4.6)	4.0 (3.2, 4.6)	4.0 (3.3, 4.6)	.936
APTT	30.5 ± 3.5	30.6 ± 3.4	30.3 ± 3.5	.401
PT	14.4 (13.6, 15.3)	14.4 (13.7, 15.2)	14.3 (13.6, 15.4)	.999
Treatment for RA ever				.897
No	77 (22.8)	39 (23.1)	38 (22.5)	
Yes	261 (77.2)	130 (76.9)	131 (77.5)	
Treatment for RA within 3 months				.231
No	163 (48.2)	87 (51.5)	76 (45)	
Yes	175 (51.8)	82 (48.5)	93 (55)	
NSAIDs, *n* (%)				.042
No	214 (63.3)	116 (68.6)	98 (58)	
Yes	124 (36.7)	53 (31.4)	71 (42)	
DMARDs, *n* (%)				.492
No	222 (65.7)	114 (67.5)	108 (63.9)	
Yes	116 (34.3)	55 (32.5)	61 (36.1)	
Hormone, *n* (%)				.167
No	224 (66.3)	118 (69.8)	106 (62.7)	
Yes	114 (33.7)	51 (30.2)	63 (37.3)	
Regular medication use *n* (%)				.154
No	189 (55.9)	101 (59.8)	88 (52.1)	
Yes	149 (44.1)	68 (40.2)	81 (47.9)	
Statins, *n* (%)				<.001
No	267 (79.0)	163 (96.4)	104 (61.5)	
Yes	71 (21.0)	6 (3.6)	65 (38.5)	
Beta‐blocker *n* (%)				.011
No	292 (86.4)	154 (91.1)	138 (81.7)	
Yes	46 (13.6)	15 (8.9)	31 (18.3)	

*Note*: Data are shown as means ± SD or median (quartile). Normal values were compared using paired and unpaired Student's *t* tests, and nonnormally distributed values were compared using Wilcoxon rank‐sum or Mann–Whitney tests.

Abbreviations: ALT, alanine transaminase; APTT, activated partial thromboplastin time; ASCVD, atherosclerotic cardiovascular disease; AST, aspartate transaminase; BMI, body mass index; CCP, anticyclic citrullinated peptide antibody; Cr, creatinine; CRP, C‐reactive protein; DD, d‐dimer; DMARDs, disease‐modifying anti‐rheumatic drugs; ESR, erythrocyte sedimentation rate; Fib, fibrinogen; HDL, high‐density lipoprotein; LDL, low‐density lipoprotein; NSAIDs, nonsteroidal antiinflammatory drugs; PLT, platelet; PT, prothrombin time; RA, rheumatoid arthritis; RF, rheumatoid factor; SD, standard deviation; TG, triglyceride.

### 3.2 Comparison of peripheral lymphocyte subsets between RA‐ASCVD and RA groups

Table 2 showed comparison of lymphocyte and T‐lymphocyte subsets between RA‐ASCVD group and RA group. In terms of CD4^+^ T cells, percentage of Th cell, Th17 cell, absolute of Th cell, Th1 cell, Th2 cell, Th17 cell, and ratio of Th/Ts, Th17/Treg were significantly higher in study group (*p* < .05). Th cells value (712 ± 333.6 vs. 804.2 ± 340.4, *p* = .012) and Th cell ratio (40.9 ± 8.4 vs. 43.6 ± 10, *p* = .008) were increased in the study group than control. And Th to Ts cells (1.6 vs. 1.9, *p* = .007) was increased similarly. Interestingly, the subsets of Th cell including Th1 (93.9 vs. 111.7, *p* = .046), Th2 (6 vs. 7, *p* = .022) and Th17(5.4 vs. 7.5, *p* < .001) were elevated in the study group, and other subsets of T cell were not significantly different, especially Treg cell, which is related to the opposite change in Th17/Treg (0.2 vs. 0.3, *p* = .001). Supporting Information: Figure 2 shows the percentage of Th17 cell, the absolute value of Th17 cell and Th17/Treg and these data were displayed in median and quartile.

**Table 2:** Comparison of lymphocyte and T‐lymphocyte subsets between RA‐ASCVD group and RA group.

Variables	Total (*n* = 338)	RA group (*n* = 169)	RA‐ASCVD group (*n* = 169)	*p* Value
T cells/μL	1253.1 ± 512.1	1201.5 ± 529.6	1304.8 ± 490.2	.064
T cells (%)	71.3 (64.3, 77.7)	70.7 (63.4, 77.0)	72.0 (65.2, 79.0)	.068
B cells/μL	166.1 (96.7, 274.4)	156.8 (95.3, 289.0)	176.0 (101.9, 264.4)	.638
B cells (%)	10.1 (6.6, 15.0)	10.3 (6.6, 15.0)	10.1 (6.8, 15.0)	.633
Th cells/μL	758.1 ± 339.7	712.0 ± 333.6	804.2 ± 340.4	.012
Th cells (%)	42.3 ± 9.3	40.9 ± 8.4	43.6 ± 10.0	.008
Ts cells/μL	421.0 (293.9, 577.9)	411.3 (293.6, 578.0)	429.7 (294.7, 577.9)	.686
Ts cells (%)	25.7 ± 8.6	26.4 ± 8.3	25.0 ± 8.9	.136
ThToTs	1.7 (1.3, 2.3)	1.6 (1.3, 2.0)	1.9 (1.4, 2.5)	.007
Nk cells/μL	237.5 (158.1, 358.4)	253.3 (158.8, 363.0)	224.8 (158.0, 350.6)	.638
Nk cells (%)	14.0 (10.0, 21.7)	15.2 (11.0, 22.9)	13.4 (9.8, 19.0)	.06
TBNk cells/μL	1754.3 ± 662.8	1717.5 ± 703.2	1791.0 ± 619.6	.309
TBNk cells (%)	98.3 (97.4, 99.0)	98.4 (97.5, 99.1)	98.0 (97.1, 99.0)	.102
Th1 cells/μL	100.3 (62.3, 164.7)	93.9 (59.7, 148.2)	111.7 (69.1, 184.6)	.046
Th1 cells (%)	15.0 (10.0, 23.0)	14.8 (9.4, 22.1)	15.5 (10.4, 24.3)	.519
Th2 cells/μL	6.6 (4.0, 10.5)	6.0 (3.8, 9.9)	7.0 (4.7, 11.8)	.022
Th2 cells (%)	1.0 (0.7, 1.5)	0.9 (0.7, 1.4)	1.0 (0.7, 1.5)	.455
Th17 cells/μL	6.3 (3.6, 10.1)	5.4 (2.8, 8.5)	7.5 (4.4, 11.0)	<.001
Th17 cells (%)	0.9 (0.6, 1.4)	0.8 (0.5, 1.3)	1.1 (0.6, 1.6)	<.001
Treg cells/μL	23.9 (16.3, 38.4)	23.2 (16.4, 34.4)	25.4 (16.3, 41.4)	.15
Treg cells (%)	3.7 (2.6, 5.2)	3.7 (2.7, 5.3)	3.6 (2.6, 5.1)	.478
Th1ToTh2	15.3 (8.8, 24.4)	16.0 (9.1, 24.7)	15.1 (8.7, 24.3)	.857
Th17ToTreg	0.2 (0.1, 0.4)	0.2 (0.1, 0.4)	0.3 (0.2, 0.5)	.001
Th1ToTreg	4.0 (2.2, 6.9)	4.0 (2.2, 6.7)	4.0 (2.3, 7.3)	.522
Th2ToTreg	0.3 (0.2, 0.4)	0.3 (0.2, 0.4)	0.3 (0.2, 0.4)	.274

*Note*: Data are shown as means ± SD or median (quartile). Normal values were compared using paired and unpaired Student's *t* tests, and nonnormally distributed values were compared using Wilcoxon rank‐sum or Mann–Whitney tests. TH/TS, which is the ratio of helper T cells to suppressor T cells and “TBNk” refers to the sum of T and B lymphocytes and natural killer cell.

Abbreviations: ASCVD, atherosclerotic cardiovascular disease; RA, rheumatoid arthritis; SD, standard deviation.

### 3.3 Univariate and multivariate analyses of factors associated with RA‐ASCVD

Logistic recession analysis was utilized to determine to relate to RA with ASCVD in Supporting Information: Table 2 and Table 3. The result of univariate logistic analysis showed that the smoking history, drinking alcohol history, body mass index (BMI), creatinine (Cr), Th/Ts, Th1 value, Th2 value, Th17 value, Th17 ratio, Treg/Th17, and NSAIDs use were significantly different with RA‐ASCVD group. The multivariate logistic analysis showed that Th/Ts, Th1 value, Th2 value, Th17 value, Th17 ratio, Th17/Treg, and Treg/Th17 were risk factors for patients with RA comorbidity with ASCVD after adjustment for sex, age, hypertension, diabetes mellitus, smoking history, drinking alcohol history, BMI, Cr, and regular medication use. Among these, Th17 cell absolute (OR = 1.11, 95% confidence interval [CI] (1.06 ~ 1.17), *p* < .001), Th17 cell ratio (OR = 1.51, 95% CI (1.08 ~ 2.11), *p* = .016), Th17/Treg (OR = 2.14, 95% CI (1.03 ~ 4.42), *p* = .041). Notably, Th17/Treg remained an independent risk factor even after adjusting for the use of drugs that significantly affect immune function.

**Table 3:** Multivariate analyses of factors associated with RA‐ASCVD.

Variable	crude. OR_95 CI	crude. *p* value	adj. OR_95 CI	adj. *p* value
ThToTs	1.38 (1.08 ~ 1.76)	.011	1.52 (1.16 ~ 1.97)	.002
Th1Value	1 (1 ~ 1.01)	.022	1 (1 ~ 1.01)	.037
Th2Value	1.05 (1.01 ~ 1.09)	.012	1.05 (1.01 ~ 1.09)	.016
Th17Value	1.11 (1.06 ~ 1.17)	<.001	1.11 (1.06 ~ 1.17)	<.001
Th17Ratio	1.53 (1.1 ~ 2.11)	.01	1.51 (1.08 ~ 2.11)	.016
Th17ToTreg	1.89 (0.94 ~ 3.81)	.073	2.14 (1.03 ~ 4.42)	.041
TregToTH17	0.91 (0.86 ~ 0.96)	.001	0.9 (0.85 ~ 0.95)	<.001

Abbreviations: ASCVD, atherosclerotic cardiovascular disease; CI, confidence interval; OR, odds ratio; RA, rheumatoid arthritis.

Model was adjusted for sex, age, hypertension, diabetes mellitus, smoking history, drinking alcohol history, BMI, Cr, and regular medication use. Regular use of hormones or antirheumatic drugs or both of two drugs by participants for the 3 months before the immune cell analysis were defined as regular medication use.

None, nonadjusted model. Model I was adjusted for age and sex htn dm. Model II was adjusted for sex, age, hypertension, diabetes mellitus, smoking history, drinking alcohol history, BMI, Cr, and regular medication use. Regular use of hormones or antirheumatic drugs or both of two drugs by participants for the 3 months before the immune cell analysis were defined as regular medication use.

Table 4 showed that logistic regression analysis of ASCVD risk for tertile 2 and 3 using tertile 1 as the control group. After the adjustment for sex, age, hypertension, diabetes mellitus, smoking history, drinking alcohol history, BMI, Cr, and regular medication use, there was a 1.75‐fold increased risk of ASCVD in the tertile 2 group (OR = 1.75, 95% CI (1.01–3.03), *p* = .048), while there was a 2.3‐fold increased risk of ASCVD in the tertile 3 group (OR = 2.3, 95% CI [1.3–4.06], *p* = .004) compared to tertile 1 group. Surprisingly, Th17/Treg remains an independent risk factor for RA‐ASCVD even after adjustment for drug use.

**Table 4:** Univariate and multivariate logistic analyses of ASCVD in tri‐sectional Th17/Treg groups.

TH17/TREG tertile	Nonadjusted	Model I	Model II
Low	1.0	1.0	1.0
Middle	1.69 (0.99, 2.87) 0.052	1.74 (1.02 ~ 2.96) 0.042	1.75 (1.01 ~ 3.03) 0.048
High	2.17 (1.27, 3.7) 0.004	2.24 (1.3 ~ 3.87) 0.004	2.3 (1.3 ~ 4.06) 0.004

Abbreviation: ASCVD, atherosclerotic cardiovascular disease.

### 3.4 Restricted cubic spline of Th17/Treg and OR of RA‐ASCVD

**Figure 1:**
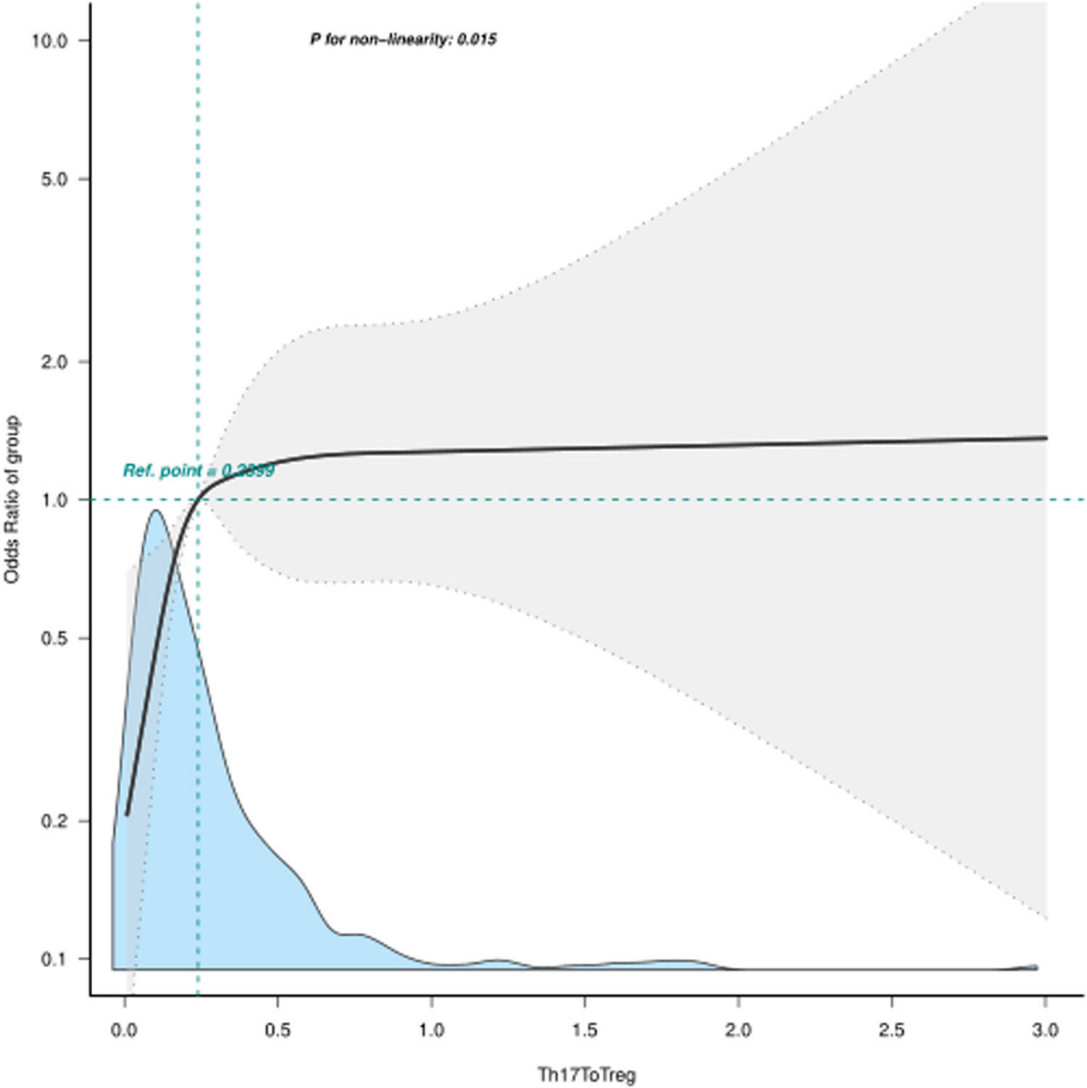
Restricted cubic spline of Th17/Treg and odds ratio of RA‐ASCVD. Model was adjusted for sex, age, hypertension, diabetes mellitus, smoking history, drinking alcohol history, BMI, Cr, and regular medication use. Regular use of hormones or antirheumatic drugs or both of two drugs by participants for the 3 months before the immune cell analysis were defined as regular medication use. ASCVD, atherosclerotic cardiovascular disease; BMI, body mass index; Cr, creatinine; RA, rheumatoid arthritis.

Figure 1 showed the restricted cubic spline of Th17/Treg and OR of RA‐ASCVD. After the adjustment for sex, age, hypertension, diabetes mellitus, smoking history, drinking alcohol history, BMI, Cr, and regular medication use, we found that the association of Th17/Treg and OR of RA‐ASCVD is nonlinear (P for nonlinearity = 0.015). Interestingly, we found a critical point of Th17/Treg (critical point = 0.2399). Th17/Treg shows a protective role in the odds of ASCVD when Th17/Treg < 0.2399. With smaller Th17/Treg, the protective efficiency is more obvious when Th17/Treg < 0.2399. When Th17/Treg > 0.2399, the odds of RA‐ASCVD are consistent statistically.

### 3.5 Subgroup analysis of Th17/Treg for RA‐ASCVD

Since some medications such as antirheumatic drugs, hormones, and statins significantly affect the levels of peripheral blood immune cell in RA patients, we conducted a subgroup analysis to investigate the effect of some drugs (NSAIDs, disease‐modifying anti‐rheumatic drugs [DMARDs], Hormone, and statin) commonly used by RA patients on the role of Th17/Treg on RA‐ASCVD. Supporting Information: Table 3 showed the effect of different medication use on the role of Th17/Treg on RA‐ASCVD. Surprisingly, the results indicated Th17/Treg was significantly affected by drug use. Only in the group not taking medications that notably affect the levels of peripheral blood immune cell, Th17/Treg is a significantly independent risk factor. Combined with Tables 3 and 4, the above results reaffirm the two facts that conventional drug therapy for RA and statin significantly affects the distribution of immune cells in the peripheral blood of RA patients and, more importantly, that Th17/Treg remains an independent risk factor after the adjustment for sex, age, hypertension, diabetes mellitus, smoking history, drinking alcohol history, BMI, Cr, and regular medication use.

## 4 DISCUSSION

In our study, Th/TS was found to be an independent risk factor for RA patients with ASCVD. This suggests that pathologically expanded peripheral T helper cells may be involved in atherosclerosis in RA patients. As we all know, CD4^+^ T cells are central mediators of autoimmune pathology. The immunogenetics of RA suggest a key role for aberrant pathways of T‐cell activation in the initiation and/or perpetuation of disease.^11^ A recent phenotypic study^12^ described the possible implication of a novel subset of peripheral T helper cells (Tph) important for T‐B cell crosstalk and plasma cell differentiation in the RA joint of ACPA+ (autoantibodies against citrullinated proteins) RA patients. T helper cells also play a vital role in atherosclerosis pathology. A large body of evidence indicates that T helper 1 (TH1) cells have proatherogenic roles and regulatory T (Treg) cells have antiatherogenic roles. Treg cells can become proatherogenic.^13^ Therefore, it is well confirmed in our study that the abnormal expansion of Th cells is related to the aggravation of RA activity. It is also related to the occurrence and development of cardiovascular complications.

Th17 cells are considered a contributing factor in the development of RA. IL‐17A produced mainly by Th17 cells is a key mediator of neutrophil activation, recruitment, and migration. Kim et al. found that Th17 cells and Th17‐related cytokines play a significant role in RA pathogenesis and that the level of Th17 cells in peripheral blood is associated with disease activity in RA.^14^ Meanwhile, rheumatologists also found that Th17 cells were heterogeneous and could differentiate into highly diverse subsets. Under different cytokines, Th17 cells gained the ability to convert toward Th1, Th2, Tfh, Treg cells, and so on. This means Th17 cells acquired pathogenic or immunosuppressive functions by differentiation in RA.^15^

As for the role of Th17 cells in atherosclerosis, they seem to have both proatherogenic and antiatherogenic functions. Some animal studies have demonstrated that IL‐17 can stabilize plaque formation by increasing type I collagen production by vascular smooth muscle cells (VSMC).^16^, ^17^ A clinical study^18^ suggested that low serum levels of IL‐17 are associated with a higher risk of major cardiovascular events in Caucasian patients with acute myocardial infarction. However, some studies have supported Th17 cells can be proatherogenic. Compared with nonatherosclerotic wild‐type littermates, Gao et al.^19^ found that Th17 cells, as well as Th1, increased in atherosclerotic ApoE^−/−^ mice. In ApoE^−/−^ mice, increased Th17 cells were associated with increased plaques. Importantly, anti–IL‐17 Ab dramatically inhibited this phenotype, whereas rIL‐17 application aggravated it. Smith et al.^20^ arrived at the same conclusion that blockade of interleukin‐17A results in reduced atherosclerosis in apolipoprotein E‐deficient mice. In some clinical studies,^21^, ^22^ consistent with some experimental studies, the elevated plasma IL‐17 level and the elevated number of peripheral Th17 cells are associated with unstable angina pectoris and acute myocardial infarction compared to stable angina and healthy individuals.

Moreover, Eid et al.^23^ demonstrated that IL‐17 is produced concomitantly with IFN‐γ by coronary artery infiltrating T cells and that these cytokines act synergistically to induce proinflammatory responses in VSMC. The role of Th17 in atherosclerosis is controversial. The reason for this phenomenon may be related to the plasticity of Th17 cells. Th17 cells acquire pathogenic or immunosuppressive functions via the differentiation activated by different cytokines. A recent single‐cell RNA‐sequencing study^24^ showed that a newly defined ApoB‐Reactive CD4^+^T‐Regulatory Cells involve in an initially protective autoimmune response against ApoB in atherosclerosis and eventually plaque T cells that expand during progression of atherosclerosis showed a mixed TH1‐/TH17 phenotype in scRNAseq. Additionally, a recent study^25^ showed that Th17/IL‐17 induces endothelial cell senescence via activation of NF‐κB/p53/Rb signaling pathway.

In our study, we found that the increase in Th17 cells' absolute number is an independent risk factor for RA patients with ASCVD. This may suggest that Th17 cells promote the occurrence and development of atherosclerosis in RA patients. Firstly, Th17 cells play a crucial role in the pathological process of RA. Pathologically increasing Th17 cells aggravate the chronic inflammatory response in RA patients by secreting inflammatory cytokines. At the same time, it promotes the formation of atherosclerotic plaques in various ways, which eventually leads to ASCVD.

It is becoming increasingly apparent to clinicians that RA is an independent risk factor for CAD, mainly because of the underlying inflammatory cascade. Anti‐inflammatory treatment has become more common. For patients with RA, biological DMARDs (tumor necrosis factor inhibitors, IL‐1 inhibitors, IL‐6 receptor inhibitors, and CD20 blockers) are used to manage atherosclerotic CVD risk and prevent atherosclerosis from forming.^5^, ^26^ Our results may imply the use of inhibitors of the IL‐17 pathway could alleviate the pathologic progress of atherosclerosis in RA patients.

In our study, we found that patients with RA‐CVD had higher Cr than patients with RA. Quite a few studies have shown that RA patients had a higher risk of developing chronic kidney disease (CKD) and glomerulonephritis. The reported kidney disease prevalence in patients with RA ranges from 5%–50% based on studies of different designs, patients with RA were more likely to develop reduced kidney function over time.^27^, ^28^, ^29^ It can affect the kidneys directly by the effect of the disease itself (presenting as secondary amyloidosis, mesangial proliferative glomerulonephritis) or secondary to the drugs used for treatment, including DMARDs or NSAIDs.^30^ There was a study show that chronically increased levels of serum amyloid A, an acute‐phase reactant precursor protein synthesized by hepatocytes, can lead to secondary amyloidosis.^31^ IL‐6 level is higher in the peripheral blood and synovia of patients with RA. In animal models, IL‐6 induced proliferation of mesangial cells in the kidneys, it may be a growth factor for mesangial cells.^32^ Similarly, the renal dysfunction and inflammation was associated with a higher risk of CVD than either condition alone in RA patients.^33^ Reduced kidney function development is enhanced in patients with RA compared to non‐RA persons and increases the risk of cardiovascular events in RA. A central mechanism in CKD involves endothelial dysfunction, and it appears to have a positive independent association with CVD in RA.^32^, ^34^ Additionally, Nordlohne et al.^35^ found that in a mouse model of atherosclerosis with moderate renal impairment, interleukin‐17 receptor A is instrumental in this condition, and blockade of this pathway can normalize arterial inflammation even in advanced atherosclerosis. It suggested that inflammation, which is a central part of the immunological pathophysiology of RA, also has a role in the development of renal dysfunction and CVDs.

Treg cells have antagonistic functions, whereas Th17 cells are pro‐inflammatory. Their respective developmental processes are linked, and Th17 and Treg cells exhibit significant adaptability. These characteristics suggest that the Th17/Treg balance is crucial to the onset and progression of RA.^36^ Wang and colleagues computed the Th17/Treg ratio in light of these conflicting findings on the frequency of Th17 and Treg cells. Th17/Treg ratios are low in healthy controls while high in RA patients, particularly those with active RA.^37^ According to a clinical investigation, the Th17/Treg imbalance may contribute to the high frequency of adverse cardiovascular events. This is done by working with microinflammation to promote immune‐mediated atherosclerosis. They believe that the development of atherosclerosis is influenced by an imbalance between pro‐ and anti‐inflammatory/atherogenic lymphocyte subpopulations.^38^ Recent research has revealed that the Th17/Treg balance may be crucial for maintaining plaque stability and controlling inflammation.^39^ Pioglitazone controls the Th17/Treg balance in an AMPK‐dependent way to demonstrate anti‐atherosclerotic effects for stabilizing atherosclerotic plaque.^40^ Angong Niuhuang Pill defends against early and midterm atherosclerotic ApoE^−/−^ mice by maintaining the Th17/Treg balance, preventing chronic inflammation, lowering the collagen content of the plaque, and decreasing inflammatory cell infiltration.^41^

This study has several weaknesses. This is a single‐center retrospective case‐control study, which cannot confirm the causal relationship between elevated Th17 cells and ASCVD in RA patients. What's more, we did not assess the severity of ASCVD due to the retrospective analysis design. This meant that we could not figure out the relationship between elevated Th17 cells and the size of atherosclerotic plaque. Last but not least, detailed drug treatment (antiplatelet agents, antirheumatic agents, etc) was not available which could affect atherosclerosis or T cell subset alteration. All these factors mentioned above should be taken into account in future studies.

## 5 CONCLUSION

This study revealed that Absolute and percentage of Th17 cells in peripheral blood of patients with RA ‐ASCVD is significantly higher. Elevated Th17/Treg may be a potential biomarker for RA patients in predicting comorbidity with ASCVD. The imbalance of Th17/Treg may be involved in the pathogenesis of ASCVD in RA. Downregulating Th17/Treg could be potential therapeutic targets for preventing RA patients from ASCVD. However, the mechanisms how elevated Th17/Treg impairs arteries and accelerates atherosclerosis in RA await further research.

## AUTHOR CONTRIBUTIONS

Bin Liang, Xin Wang, and Hongxuan Fan study design. Yongle Wang, Miao Wang, and Jianqi Zhao data analysis. Hongxuan Fan, Shaobin Mao, and Jianqi Zhao article drafting. Hongxuan Fan and Jianqi Zhao data collection. Caihong Wang article revision. Bin Liang review and final approval. All authors contributed to the article and approved the submitted version.

## Supporting information

Supporting information.

## Data Availability

The original contributions presented in the study are included in the article. Further inquiries can be directed to the corresponding authors.

## References

[1] Sparks JA . Rheumatoid arthritis. Ann Intern Med. 2019;170(1):ITC1‐ITC16. 30596879 10.7326/AITC201901010

[2] Lai NL , Jia W , Wang X , et al. Risk factors and changes of peripheral NK and T cells in pulmonary interstitial fibrosis of patients with rheumatoid arthritis. Can Respir J. 2019;2019:1‐6. 10.1155/2019/7262065 PMC6914899 31885749

[3] Houri Levi E , Watad A , Whitby A , et al. Coexistence of ischemic heart disease and rheumatoid arthritis patients—Ā case control study. Autoimmun Rev. 2016;15(4):393‐396. 26808075 10.1016/j.autrev.2016.01.006

[4] Wang X , Fan H , Wang Y , et al. Elevated peripheral T helper cells are associated with atrial fibrillation in patients with rheumatoid arthritis. Front Immunol. 2021;12:744254. 34721413 10.3389/fimmu.2021.744254 PMC8554094

[5] Semb AG , Ikdahl E , Wibetoe G , Crowson C , Rollefstad S . Atherosclerotic cardiovascular disease prevention in rheumatoid arthritis. Nat Rev Rheumatol. 2020;16(7):361‐379. 32494054 10.1038/s41584-020-0428-y

[6] Zhao D , Liu J , Wang M , Zhang X , Zhou M . Epidemiology of cardiovascular disease in China: current features and implications. Nat Rev Cardiol. 2019;16(4):203‐212. 30467329 10.1038/s41569-018-0119-4

[7] Rahman MS , Woollard K . Atherosclerosis. Adv Exp Med Biol. 2017;1003:121‐144. 28667557 10.1007/978-3-319-57613-8_7

[8] Wilson PWF , Polonsky TS , Miedema MD , Khera A , Kosinski AS , Kuvin JT . Systematic review for the 2018 AHA/ACC/AACVPR/AAPA/ABC/ACPM/ADA/AGS/APhA/ASPC/NLA/PCNA guideline on the management of blood cholesterol: areport of the American College of Cardiology/American Heart Association Task Force on Clinical Practice Guidelines. Circulation. 2019;139(25):e1187. 10.1161/CIR.0000000000000700 30586775

[9] Gisterå A , Hansson GK . The immunology of atherosclerosis. Nat Rev Nephrol. 2017;13(6):368‐380. 28392564 10.1038/nrneph.2017.51

[10] Arnett FC , Edworthy SM , Bloch DA , et al. The American Rheumatism Association 1987 revised criteria for the classification of rheumatoid arthritis. Arthritis Rheum. 1988;31(3):315‐324. 3358796 10.1002/art.1780310302

[11] Cope AP , Schulze‐Koops H , Aringer M . The central role of T cells in rheumatoid arthritis. Clin Exp Rheumatol. 2007;25(5 suppl 46):S4‐S11. 17977483

[12] Rao DA , Gurish MF , Marshall JL , et al. Pathologically expanded peripheral T helper cell subset drives B cells in rheumatoid arthritis. Nature. 2017;542(7639):110‐114. 28150777 10.1038/nature20810 PMC5349321

[13] Saigusa R , Winkels H , Ley K . T cell subsets and functions in atherosclerosis. Nat Rev Cardiol. 2020;17(7):387‐401. 32203286 10.1038/s41569-020-0352-5 PMC7872210

[14] Kim J , Kang S , Kim J , Kwon G , Koo S . Elevated levels of T helper 17 cells are associated with disease activity in patients with rheumatoid arthritis. Ann Lab Med. 2013;33(1):52‐59. 23301223 10.3343/alm.2013.33.1.52 PMC3535197

[15] Yang P , Qian FY , Zhang MF , et al. Th17 cell pathogenicity and plasticity in rheumatoid arthritis. J Leukoc Biol. 2019;106(6):1233‐1240. 31497905 10.1002/JLB.4RU0619-197R

[16] Brauner S , Jiang X , Thorlacius GE , et al. Augmented Th17 differentiation in Trim21 deficiency promotes a stable phenotype of atherosclerotic plaques with high collagen content. Cardiovasc Res. 2018;114(1):158‐167. 29016728 10.1093/cvr/cvx181

[17] Gisterå A , Robertson AKL , Andersson J , et al. Transforming growth factor‐β signaling in T cells promotes stabilization of atherosclerotic plaques through an interleukin‐17‐dependent pathway. Sci Transl Med. 2013;5(196):196ra100. 10.1126/scitranslmed.3006133 23903754

[18] Simon T , Taleb S , Danchin N , et al. Circulating levels of interleukin‐17 and cardiovascular outcomes in patients with acute myocardial infarction. Eur Heart J. 2013;34(8):570‐577. 22956509 10.1093/eurheartj/ehs263

[19] Gao Q , Jiang Y , Ma T , et al. A critical function of Th17 proinflammatory cells in the development of atherosclerotic plaque in mice. J Immunol. 2010;185(10):5820‐5827. 20952673 10.4049/jimmunol.1000116 PMC12230985

[20] Smith E , Prasad KMR , Butcher M , et al. Blockade of interleukin‐17A results in reduced atherosclerosis in apolipoprotein E‐deficient mice. Circulation. 2010;121(15):1746‐1755. 20368519 10.1161/CIRCULATIONAHA.109.924886 PMC2929562

[21] Cheng X , Yu X , Ding Y , et al. The Th17/Treg imbalance in patients with acute coronary syndrome. Clin Immunol. 2008;127(1):89‐97. 18294918 10.1016/j.clim.2008.01.009

[22] Hashmi S , Zeng QT . Role of interleukin‐17 and interleukin‐17‐induced cytokines interleukin‐6 and interleukin‐8 in unstable coronary artery disease. Coron Artery Dis. 2006;17(8):699‐706. 17119379 10.1097/01.mca.0000236288.94553.b4

[23] Eid RE , Rao DA , Zhou J , et al. Interleukin‐17 and Interferon‐γ are produced concomitantly by human coronary artery–Infiltrating T cells and act synergistically on vascular smooth muscle cells. Circulation. 2009;119(10):1424‐1432. 19255340 10.1161/CIRCULATIONAHA.108.827618 PMC2898514

[24] Wolf D , Gerhardt T , Winkels H , et al. Pathogenic autoimmunity in atherosclerosis evolves from initially protective apolipoprotein B100 –reactive CD4+ T‐regulatory cells. Circulation. 2020;142(13):1279‐1293. 32703007 10.1161/CIRCULATIONAHA.119.042863 PMC7515473

[25] Zhang L , Liu M , Liu W , et al. Th17/IL‐17 induces endothelial cell senescence via activation of NF‐κB/p53/Rb signaling pathway. Lab Invest. 2021;101(11):1418‐1426. 34172831 10.1038/s41374-021-00629-y

[26] Wang L , Zhang Y , Zhang SY . Immunotherapy for the rheumatoid arthritis‐associated coronary artery disease: promise and future. Chin Med J. 2019;132(24):2972‐2983. 31855971 10.1097/CM9.0000000000000530 PMC6964948

[27] Oweis AO , Alawneh KM , Alshelleh SA , Alnaimat F , Alawneh D , Zahran DJ . Renal dysfunction among rheumatoid arthritis patients: a retrospective cohort study. Ann Med Surg. 2020;60:280‐284. 10.1016/j.amsu.2020.11.011 PMC7649584 33204418

[28] Chiu HY , Huang HL , Li CH , et al. Increased risk of chronic kidney disease in rheumatoid arthritis associated with cardiovascular complications—A national population‐based cohort study. PLoS One. 2015;10(9):e0136508. 26406879 10.1371/journal.pone.0136508 PMC4583248

[29] Hickson LJ , Crowson CS , Gabriel SE , McCarthy JT , Matteson EL . Development of reduced kidney function in rheumatoid arthritis. Am J Kidney Dis. 2014;63(2):206‐213. 24100126 10.1053/j.ajkd.2013.08.010 PMC3944015

[30] Kapoor T , Bathon J . Renal manifestations of rheumatoid arthritis. Rheumatic Disease Clinics of North America. 2018;44(4):571‐584. 30274624 10.1016/j.rdc.2018.06.008

[31] Chovanova L , Vlcek M , Krskova K , et al. Increased production of IL‐6 and IL‐17 in lipopolysaccharide‐stimulated peripheral mononuclears from patients with rheumatoid arthritis. Gen Physiol Biophys. 2014;32(3):395‐404. 10.4149/gpb_2013043 23817641

[32] Amann K , Wanner C , Ritz E . Cross‐talk between the kidney and the cardiovascular system. JASN. 2006;17(8):2112‐2119. 16825329 10.1681/ASN.2006030204

[33] Kochi M , Kohagura K , Shiohira Y , Iseki K , Ohya Y . Chronic kidney disease, inflammation, and cardiovascular disease risk in rheumatoid arthritis. J Cardiol. 2018;71(3):277‐283. 28969969 10.1016/j.jjcc.2017.08.008

[34] Dessein PH , Hsu HC , Tsang L , et al. Kidney function, endothelial activation and atherosclerosis in black and white Africans with rheumatoid arthritis. PLoS One. 2015;10(3):e0121693. 25806966 10.1371/journal.pone.0121693 PMC4373952

[35] Nordlohne J , Helmke A , Ge S , et al. Aggravated atherosclerosis and vascular inflammation with reduced kidney function depend on Interleukin‐17 receptor A and are normalized by inhibition of Interleukin‐17A. JACC Basic Transl Sci. 2018;3(1):54‐66. 30062194 10.1016/j.jacbts.2017.08.005 PMC6058956

[36] Noack M , Miossec P . Th17 and regulatory T cell balance in autoimmune and inflammatory diseases. Autoimmun Rev. 2014;13(6):668‐677. 24418308 10.1016/j.autrev.2013.12.004

[37] Wang W , Shao S , Jiao Z , Guo M , Xu H , Wang S . The Th17/Treg imbalance and cytokine environment in peripheral blood of patients with rheumatoid arthritis. Rheumatol Int. 2012;32(4):887‐893. 21221592 10.1007/s00296-010-1710-0

[38] Potekhina AV , Pylaeva E , Provatorov S , et al. Treg/Th17 balance in stable CAD patients with different stages of coronary atherosclerosis. Atherosclerosis. 2015;238(1):17‐21. 25461734 10.1016/j.atherosclerosis.2014.10.088

[39] He X , Liang B , Gu N . Th17/Treg imbalance and atherosclerosis. Dis Markers. 2020;2020:1‐8. 10.1155/2020/8821029 PMC7648711 33193911

[40] Tian Y , Chen T , Wu Y , et al. Pioglitazone stabilizes atherosclerotic plaque by regulating the Th17/Treg balance in AMPK‐dependent mechanisms. Cardiovasc Diabetol. 2017;16(1):140. 29084546 10.1186/s12933-017-0623-6 PMC5663071

[41] Fan Q , Liu Y , Rao J , et al. Anti‐atherosclerosis effect of Angong Niuhuang Pill via regulating Th17/Treg immune balance and inhibiting chronic inflammatory on ApoE(‐/‐) mice model of early and mid‐term atherosclerosis. Front Pharmacol. 2020;10:1584. 32082145 10.3389/fphar.2019.01584 PMC7005527

