# Supplementary material for: Circulating Th17/Treg as a promising biomarker for patients with rheumatoid arthritis in indicating comorbidity with atherosclerotic cardiovascular disease
Source: Clin Cardiol. 2023 Sep 4;46(12):1519–29. doi: 10.1002/clc.24065 (PMC10716320; doi:10.1002/clc.24065)
Supplement: Supplementary file 1 — Supporting information. [file CLC-46-1519-s001.docx]

**Supplementary Figure 1** shows the flow chart of screening and enrollment, which eventually included 169 RA-ASCVD cases and 169 RA controls.


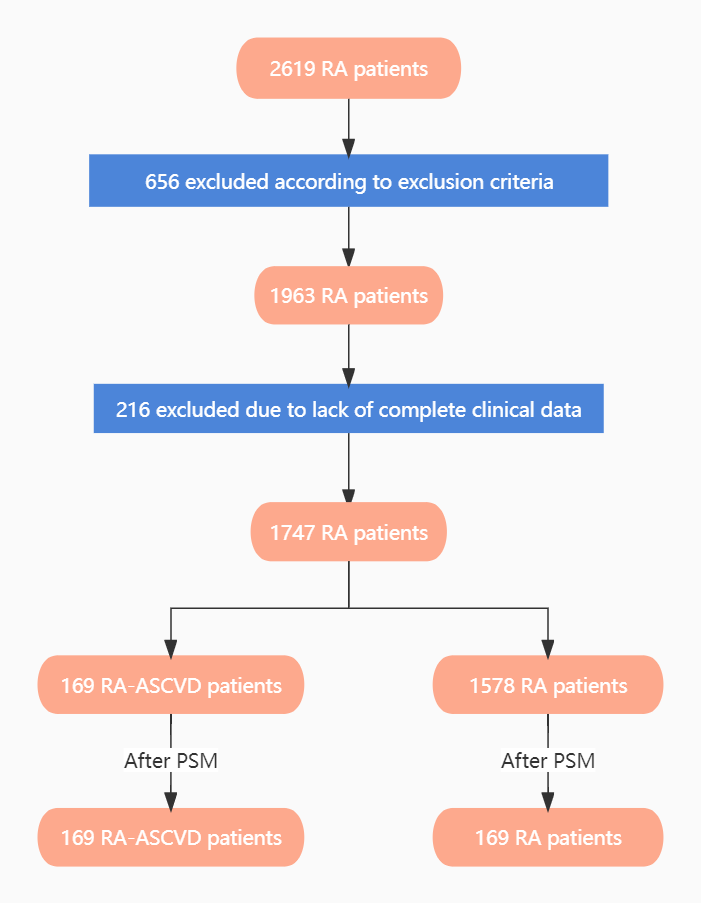


| **Supplementary Table1** \| Baseline characteristics of patients with PSM | | | | | | |
| --- | --- | --- | --- | --- | --- | --- |
|  | Before PSM | | | After PSM | | |
|  | RF-ASCVD(n=169) | Control(n=1578) | P | RF-ASCVD(n=169) | Control(n=169) | P |
| Age | 63.9±8.4 | 53.9±13.0 | 0.001 | 63.9±8.4 | 64.7±8.2 | 0.38 |
| Sex | 71(42%) | 461(29.1%) | ＜0.000 | 71(42%) | 104 (61.5) | 0.579 |
| HTN | 76(45%) | 346(21.9%) | ＜0.000 | 76(45%) | 80 (47.3) | 0.743 |
| DM | 128(75.7%) | 153(9.7%) | ＜0.000 | 128(75.7%) | 128 (75.7) | 1 |

HTN refers to hypertension and DM refers to diabetes mellitus.


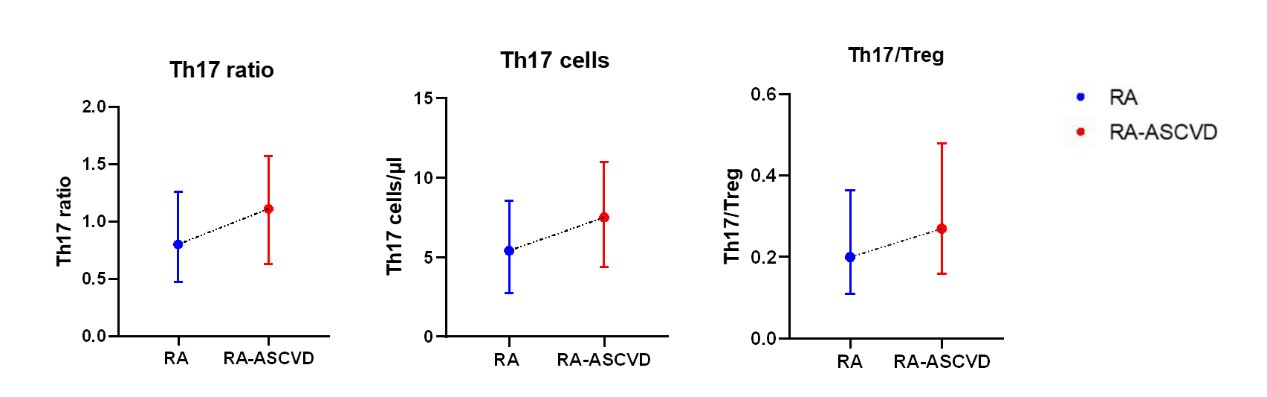


**Supplementary Figure 2** shows that the value of Th cell, Th1 cell and Th17 cell is displayed in median and quartile. The dots represent median and the upper and lower lines represent quartile. RA group is indicated in blue and RA-ASCVD group is indicated in red.

**Supplementary Table 2** Univariate analyses of factors associated with RA-ASCVD

Variable OR_95CI P_value

smoking 1.71 (1.07~2.73) 0.025

drinking 1.92 (1.04~3.53) 0.037

BMI 1.1 (1.03~1.17) 0.004

Cr 1.02 (1~1.04) 0.018

ThToTs 1.38 (1.08~1.76) 0.011

Th1Value 1 (1~1.01) 0.022

Th2Value 1.05 (1.01~1.09) 0.012

Th17Value 1.11 (1.06~1.17) <0.001

Th17Ratio 1.53 (1.1~2.11) 0.01

Th17/Treg 1.89 (0.94~3.81) 0.073

Treg/Th17 0.91 (0.86~0.96) 0.001

Treatment for RA ever 1.03 (0.62~1.72) 0.897

Treatment for RA within 3 month 1.3 (0.85~1.99) 0.231

NSAIDs 1.59 (1.02~2.48) 0.043

DMARDs 1.17 (0.75~1.84) 0.492

hormone 1.38 (0.87~2.16) 0.168

Regular medication use 1.37 (0.89~2.1) 0.155

Regular use of hormones or antirheumatic drugs or both of two drugs by participants for the 3 months prior to the immune cell analysis were defined as regular medication use.

**Supplementary Table 3** Subgroup analysis of Th17/Treg for RA-ASCVD

| Subgroup | adj.OR_95CI | adj.P_value | P.for.interaction |
| --- | --- | --- | --- |
| Treatment for RA within 3 month |  |  |  |
| No | 8.98 (1.98~40.65) | 0.004 | 0.027 |
| Yes | 1.02 (0.41~2.56) | 0.968 |  |
| NSAIDs |  |  |  |
| No | 5.76 (1.81~18.35) | 0.003 | 0.009 |
| Yes | 0.6 (0.18~2) | 0.402 |  |
| DMARDs |  |  |  |
| No | 2.69 (1.09~6.64) | 0.032 | 0.607 |
| Yes | 1.4 (0.37~5.31) | 0.62 |  |
| HORMONE |  |  |  |
| No | 3.7 (1.3~10.56) | 0.015 | 0.15 |
| Yes | 1 (0.32~3.12) | 0.994 |  |
| Regular medication use |  |  |  |
| No | 3.79 (1.18~12.19) | 0.025 | 0.195 |
| Yes | 1.19 (0.43~3.26) | 0.736 |  |
| Stations |  |  |  |
| No | 2.18 (1.02~4.63) | 0.043 | 0.492 |
| Yes | 2.21 (0.02~325.63) | 0.755 |  |

Model was adjusted for sex, age, hypertension, diabetes mellitus, somking history, drinking alcohol history, BMI and Cr. Regular use of hormones or antirheumatic drugs or both of two drugs by participants for the 3 months prior to the immune cell analysis were defined as regular medication use.
